# Ubiquitin Proteasome pathway proteins as potential drug targets in parasite Trypanosoma cruzi

**DOI:** 10.1038/s41598-018-26532-z

**Published:** 2018-05-30

**Authors:** Ishita Gupta, Suruchi Aggarwal, Kanika Singh, Amit Yadav, Sameena Khan

**Affiliations:** 1 Structural Immunology Group, International Centre for Genetic Engineering and Biotechnology, New Delhi, 110067 India; 2 Drug Discovery Research Centre, Translational Health Science and Technology Institute, Faridabad, 121001 India

## Abstract

Trypanosomiasis infects more than 21 million people and claims approximately 2 million lives annually. Due to the development of resistance against currently available anti-trypanosomal drugs, there is a growing need for specific inhibitors and novel drug targets. Of late, the proteins from the Ubiquitin Proteasome Pathway (UPP): ubiquitin ligases and deubiquitinase have received attention as potential drug targets in other parasites from the apicomplexan family. The completion of *Trypanosoma cruzi* (Tc) genome sequencing in 2005 and subsequent availability of database resources like TriTrypDB has provided a platform for the systematic study of the proteome of this parasite. Here, we present the first comprehensive survey of the UPP enzymes, their homologs and other associated proteins in trypanosomes and the UPPs from *T. cruzi* were explored in detail. After extensive computational analyses using various bioinformatics tools, we have identified 269 putative UPP proteins in the *T. cruzi* proteome along with their homologs in other *Trypanosoma* species. Characterization of *T. cruzi* proteome was done based on their predicted subcellular localization, domain architecture and overall expression profiles. Specifically, unique domain architectures of the enzymes and the UPP players expressed exclusively in the amastigote stage provide a rationale for designing inhibitors against parasite UPP proteins.

## Introduction

**Figure 1:**
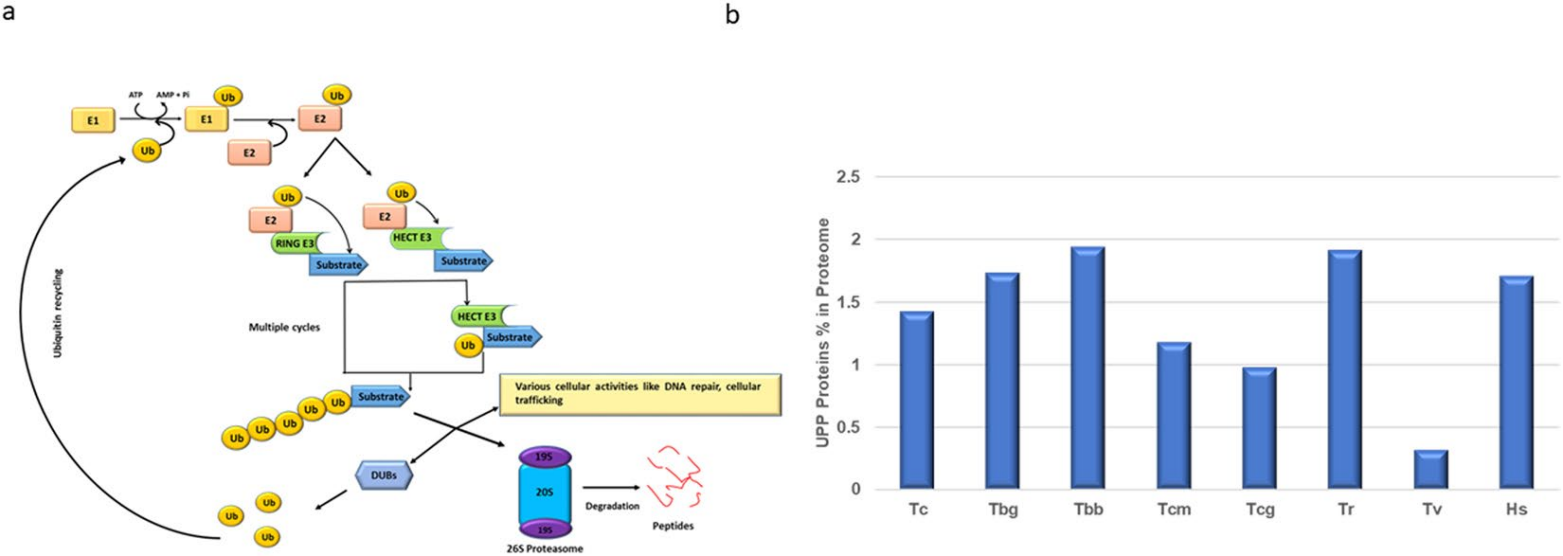
(**a**) The Schematic of the Ubiquitin Proteasome Pathway (UPP). Ubiquitination is achieved by sequential reactions involving E1s, E2s, E3s, and DUBs. Ubiquitin is activated by ubiquitin-activating enzymes (E1s) and transferred to ubiquitin-conjugating enzymes (E2s) and transferred to substrate with specific ubiquitin ligases (E3s). E3s are grouped into two categories- RING and HECT. RING class transfer ubiquitin directly to a lysine residue of protein and HECT class transfer first to HECT domain of HECT E3 ligase and then to a lysine residue of the target protein. Repeated conjugation generates polyubiquitination chain that can serve as a signal for degradation by the 26S proteasome or regulate various function depending upon lysine residue linked to polyubiquitin chain. Deubiquitination enzymes(DUBs) recycle the ubiquitin. (**b**) Percentage of proteome involved in UPP. The vertical axis shows the percentage of proteome involved in UPP machinery and the horizontal axis shows the name of species. Abbreviations for species are: *Trypanosoma cruzi* (Tc)*, Trypanosoma brucei gambiense*(Tbc), *Trypanosoma brucei brucei* (Tbb), *Trypanosoma cruzi marinkellei* (Tcm), *Trypanosoma congolense* (Tcg), *Trypanosoma rangeli* (Tr), *Trypanosoma vivax* (Tv), *Homo sapiens* (Hs).

Ubiquitin (Ub) is a protein of 76-amino acid residue exhibiting high sequence conservation among eukaryotes. Ubiquitination is the post-translational addition of ubiquitin to target proteins to control their intracellular levels through proteasome-mediated proteolysis as well as to modulate their functions by proteasome independent processes. Another class of proteins related to Ub called as ubiquitin like modifiers (Ubls), which shares high structural similarity with the Ub fold^1^. Most common Ubls found in *Homo sapiens* are ISG15, Atg8/atg12, Urm1, Ufm1, SUMO, NEDD8, Fat10, Hub1 and FUBI/MNSFβ^2^. In the first step, the carboxyl group of glycine of Ub/Ubl is activated by E1. In this process, the catalytic cysteine residue of E1 gets attached to the Ub/Ubl by a thioester bond with the release of AMP. In the second step, an activated Ub/Ubl is transferred to ubiquitin conjugating enzymes (E2s) by transacylation reaction. In the last step, E2 transfers the Ub/Ubl to protein substrate with the assistance of target specific classes of ubiquitin ligases (E3s), accompanied by the formation of an isopeptide bond between carboxyl group of glycine and ϵ-amino group of a lysine residue on the substrate protein (see Fig. 1a).

E3 ligases are classified into three major classes: HECT, RING and RBR based on their characteristic domains and mechanism of ubiquitin transfer to substrate. For HECT E3 ligases, the Ub/Ubl is transferred to E3 and forms a thioester intermediate; but in case of RING and U-box E3 ligases, the Ub/Ubl is directly transferred to substrate^3–5^. RING E3 ligases can exist as single chain enzymes or form multi subunit complexes such as Cullin ligases where it associates with F-box proteins that help in substrate recognition. RING E3 ligases forms the largest group and are characterized by conserved cysteine and histidine residues in their core domain that promote protein–protein interactions^6^. RBR have the presence of two predicted RING domains (RING1 and RING2) separated by an in-between-RING domain (IBR). After monoubiquitination, the same conjugation cascade forms a polyubiquitin chain by subsequent iterative additions of ubiquitin moieties. This process ultimately targets the substrate protein to the 26S proteasome for destruction. The specificity of substrates to be ubiquitinated is determined exclusively by a single E3 enzyme or E3 multiprotein complexes thus resulting in selective degradation of proteins. Deubiquitinating enzymes (DUBs) are proteases that reverse the effect of E3s. DUBs perform a nucleophilic attack on the carbonyl group of Ub- substrate isopeptide bond. The removal of ubiquitin chains results in reversal of signaling or rescue of protein from degradation, or the recycling of ubiquitin for ubiquitin homeostasis (see Fig. 1a). DUBs are classified into five classes - aspartic, metallo, serine, threonine, and cysteine proteases, based on residues responsible for catalytic activity. Cysteine proteases are further classified into several subclasses like- ubiquitin-specific protease (USP), ubiquitin C-terminal hydrolase (UCH), otubain protease (OTU), and machado-joseph disease protease (MJD)^7^. Metallo-proteases are JAMM (JAB1/MPN/Mov34 metalloenzyme) proteases, which use metal ions for catalysis. Cysteine proteases contain catalytic triads in which the deprotonation of catalytic cysteine is carried out by a histidine residue, which is polarized by an aspartic acid residue. In, metallo proteases, a zinc atom is stabilized by an aspartate, two histidines and a water molecule to form an intermediate with the substrate^7^. Any abnormality in the functioning of the UPP proteins can result in the impairment of cellular homeostasis and lead to several disorders such as cardiovascular diseases, neurodegenerative diseases, malignancies and systemic auto immunity^8–11^.

The ubiquitination system is indispensable not just to higher eukaryotes, but also unicellular parasitic protozoans such as *Trypanosoma*. Trypanosomiasis is a neglected tropical deadly disease caused by different species of *Trypanosoma* affecting humans and other animals. *T. cruzi* causes the Chagas disease with high mortality and morbidity in humans^12^. The life cycle of this parasite shuttles between a hematophagous triatomine insect and a vertebrate host^13^. Currently, there are no vaccines available to treat this disease^14^. There are two first line drugs benznidazole and nifurtimox available in most countries to treat the Chagas disease, but their use in chronic phases are controversial^15^. There is an unmet need to discover new therapies and drug targets to overcome the disease as existing therapies are insufficient and underdeveloped. The availability of genomic and proteomic data for *T. cruzi* will help in the identification of new drug targets.

The ubiquitin proteasome pathway enzymes, versatile players of the protein degradation machinery, have recently started receiving attention in the kinetoplastids from a drug target discovery perspective. Studies supported that the *T. cruzi* ubiquitin protein is different from its human homolog by only three amino acids but the antibodies present in sera from Chagas patients are specific to *T. cruzi* only^16,17^. The 26S proteasome was identified in the epimastigote stage of Trypanosoma as a high molecular weight complex (1,400 kDa) with a composition similar to the canonical eukaryotic proteasome^18^. The role of the proteasome is indicated by the presence of increased level of ubiquitinated protein when trypomastigotes are transformed into amastigotes. During trypomastigote-to-amastigote transformation, the cystoskeletal proteins associated with the flagellum (paraflagellar rod proteins), are shown to be degraded by the ubiquitin proteasome pathway^19^. The proteasome inhibitors lactacystin and MG132 inhibit the transformation of trypomastigotes into amastigotes^20^. Clasto lactacystin, an inactive analogue of lactacystin, and cell-permeant peptide aldehyde inhibitors of *T. cruzi* cysteine proteinases were shown to have no effect^20^. This indicated that protein degradation that occurs during parasite cell differentiation is primarily proteasome dependent. Trypanosoma proteasomes are localized in nucleus, cytoplasm and kinetoplast, suggesting an important role of ubiquitin proteasome system in kinetoplast biochemistry. Proteasome inhibition in epimastigote results in an increase of oxidized protein levels, demonstrating the role of proteasomes in transformation of non-infectious epimastigotes to non-replicative and infectious trypomastigotes^21^. *In vitro* metacyclogenesis is strongly (95%) inhibited by 5 µM lactacystin treatment. Epimastigotes treated with proteasome inhibitor do not block cell adhesion but are not able to differentiate into metacyclic trypomastigotes. This finding revealed that proteasomal proteolysis occurs during metacyclogenesis^21,22^. Here in this study, we have catalogued and characterized *T. cruzi* UPP enzymes using bioinformatics approaches to predict their domain architecture and, localization and, pulled out unique sets of enzymes that can function as a potential drug targets.

## Results and Discussion

### Sequence extraction and analysis of Ubiquitin Proteasome pathway (UPP) components in Trypanosoma genomes

We downloaded the current Uniprot version and used Hidden Markov Models (HMMs) to identify the repertoire of UPP proteins in the translated genomes of *T. cruzi* (Tc)*, T. brucei gambiense* (Tbg), *T. brucei brucei* (Tbb), *T. cruzi marinkelli* (Tcm), *T. congolense* (Tcg), *T. rangeli* (Tr)*, T. vivax (*Tv*) and* the host *Homo sapiens* (Hs). We have used HMMer tool based on Hidden Markov Models (HMMs) for identifying the common domains (24 Pfam domains) present in UPP proteins such as Ubiquitin (Ub), Ubiquitin like modifiers (Ubls), Ubiquitin activating enzymes (E1s), Ubiquitin conjugating enzymes (E2s), Ubiquitin ligases (E3s) and Deubiquitinating enzymes (DUBs). The cutoff for the HMM searches were evaluated using a series of thresholds ranging from e-value 0.1, 0.2 to 1.0. We observed that at all the thresholds, there was no difference in the number of hits identified, suggesting that the homologs identified are not sensitive to tweaks in the score threshold and any score cutoff will lead to the same robust results. This was critical to establish that the hits were invariant at increasing thresholds and ensure that downstream inferences are reasonable. In this case, due to no difference between thresholds, results from the e-value 0.1 cutoff was selected for further analysis. A total of 1,229 UPPs in *H. sapiens* have been analyzed previously with a cutoff of 0.5 e-value using HMM search^23^. While we report 1,227 UPP proteins in *H. Sapiens* (see Table 1), this is consistent with reported data sets. Furthermore, the Hidden Markov Model that we used to search for UPP has also demonstrated its applicability to the detailed identification of apicomplexan UPP pathway proteins^24^.

**Table 1:** Predicted number of UPP components in 8 analyzed *genomes*.

HMM	Tc	Tbg	Tbb	Tcm	Tcg	Tr	Tv	Hs
***Ubiquitin and ubiquitin like proteins***								
Ubiquitin*	31	16	18	1	4	20	2	45
UFM1	2	1	1	0	0	0	0	1
URM1	2	1	1	0	0	0	0	3
ATG8	3	3	3	0	0	0	0	15
APG12	1	3	3	0	0	0	0	1
***Ubiquitin-activating enzymes (E1)***								
THIF	23	12	12	12	5	13	1	42
***Ubiquitin-conjugating enzymes (E2)***								
UQ_con	29	17	17	9	4	14	1	87
***Ubiquitin ligases (E3)***								
CULLIN	13	8	7	8	5	7	1	16
UBOX	5	4	4	4	0	2	0	20
FBOX	8	11	9	9	2	6	0	111
HECT	16	11	11	12	7	13	0	45
RING	67	44	44	30	13	33	5	631
***Deubiquitinases (DUBs)***								
WLM	2	1	1	1	1	1	0	0
OTU	8	4	4	4	0	4	0	21
JOSEPHIN	0	0	0	0	0	0	0	13
Peptidase_C48	3	1	1	2	2	1	0	16
Peptidase_C54	2	2	2	2	1	2	0	7
Peptidase_C97	10	6	6	5	3	3	0	8
UCH*	31	16	15	16	8	15	1	123
JAB	14	5	6	4	3	6	1	17
Ribosomal_S19e	2	2	2	0	0	1	0	5
*Total*	**269**	**168**	**167**	**119**	**58**	**141**	**12**	**1227**

The number of UPP components identified in *Trypansoma cruzi* is highest among all species of parasite. Symbol - * indicates that three of the Uniprot Ids are in both Ubiquitin category of Ubiquitin and ubiquitin-like proteins and UCH category of Deubiquitinases (Q4CYB0, Q4CL15, Q4D313). Abbreviations used: *Trypanosoma cruzi* (Tc), *Trypanosoma brucei gambiense* (Tbg), *Trypanosoma brucei brucei* (Tbb), *Trypanosoma cruzi marinkellei* (Tcm), *Trypanosoma congolense* (Tcg), *Trypanosoma rangeli* (Tr), *Trypanosoma vivax* (Tv), Homo *sapiens* (Hs).

To carry out a comparative analysis of UPP proteins of *T. cruzi*, we considered UPP protein sequences from its host *H. sapiens*. As expected, we found variable numbers of UPP proteins in different species of Trypanosoma and in *H. sapiens*. In each case, these numbers of UPP proteins represent approximately 1.39% of their respective proteomes (see Fig. 1b). The Trypanosoma genome have tendency to exhibit functional redundancy, manifested as multiple isoforms of the same enzyme. There is a possibility that sequences of other species that have not yet been annotated show a different proportion of UPP proteins. We also noticed that the number of individual UPP proteins varies in different species. *T. congolense and T. vivax* have the lowest UPP proteins count amongst other *Trypanosoma* species and *H. sapiens*. The low or variable UPP numbers in different *Trypanosoma* species may suggest the incompleteness of their respective genome projects. This leads to a conditional reduction in UPP components. *H. sapiens* contains the highest number of UPP proteins in this analysis (see Table 1). With regards to the relative abundance of each domain family, a striking observation is that a high proportion of RING E3 ligases (61.46%) and UCH containing DUBs proteins (43.05%) are present in *T. cruzi*, while only a few of them were identified belong to other E3s and DUBs. This indicates these proteins possibly have many interacting partners and are involved in diverse cellular functions.

### Comparative Genomic analysis of Ubiquitin Proteasome pathway (UPP) components in Trypanosoma genomes

**Figure 2:**
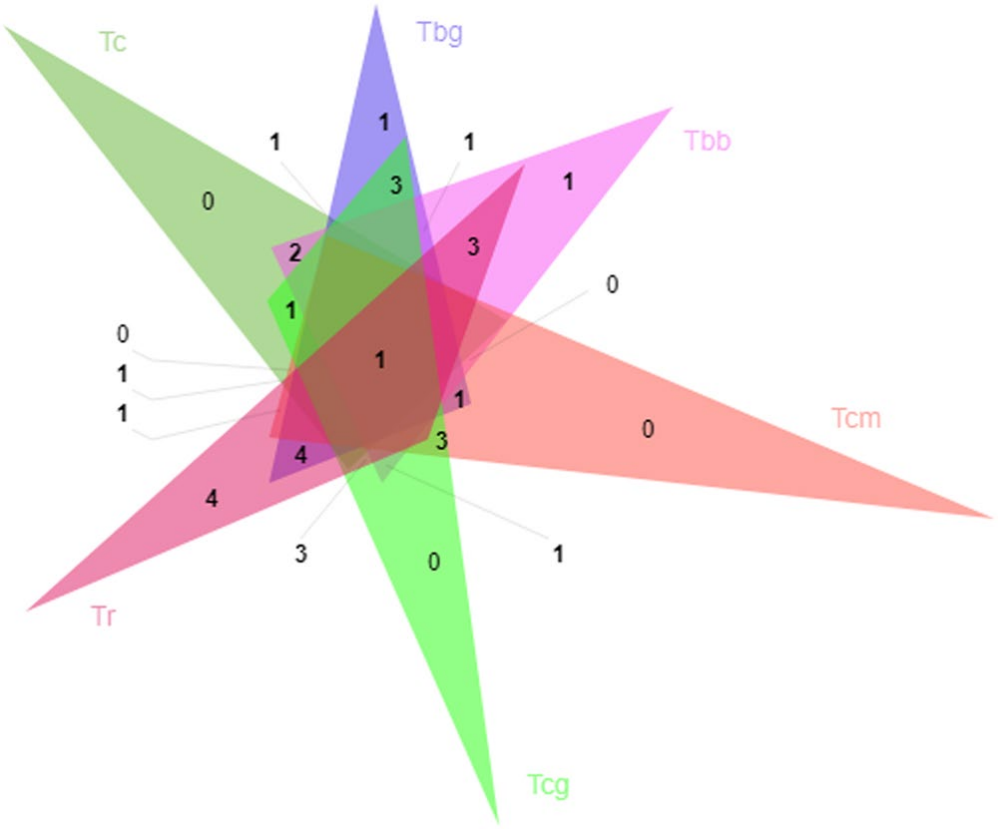
Comparitive analysis of UPP proteins orthologous clusters. The orthologous clustrers was generated by OrthoVenn of species *T. cruzi* (Tc)*, T. brucei gambiense* (Tbg), *T. brucei brucei* (Tbb), *T. cruzi marinkelli* (Tcm), *T. congolense* (Tcg), *T. rangeli* (Tr). The number in Venn diagram represents the unique and shared orthologous proteins of each species.

A comparative analysis to identify orthologous proteins among Trypanosoma genomes was performed using OrthoVenn. For the whole genome sets, we identified more than 9059 clusters. As many of the clusters (8870) were singletons (occurring in a single species), we were not interested in pursuing those for further analysis. As our focus was on UPPs, we analyzed and mapped the corresponding orthologs of 269 proteins we filtered in our initial analysis from *T. cruzi*. We observed that out of 269, 6 proteins from *T.cruzi* had no ortholog in other species. Of these, four proteins (Q4CL15, Q4CXY4, Q4CUI7 and Q4D018) are known to be involved in protein deubiquitination and ubiquitin-dependent protein catabolic process. One of these proteins (Q4CK94) is involved in ubiquitin-protein transferase activity and the other (Q4CQE9) is an uncharacterized protein, with unknown function but contains a ubiquitin domain. These six proteins may be responsible for differential pattern of ubiquitination in this species. We also analyzed the domains observed in hmmer search results with respect to the orthologs of the 269 target proteins of *T. cruzi*. We observed that a lot of proteins that were observed as orthologs were not identified in hmm search (see Supplementary Table 1). This could be because these proteins may have sequence similarity but lost UPP domains over the course of time. We then compared the proteins only identified by hmm search for six species using OrthoVenn. The analysis showed that 46 orthologous clusters were found commonly in *T. cruzi, T. brucei gambiense*, *T. brucei brucei*, *T. cruzi marinkelli, T. congolense* and *T. rangeli*. The number in Venn diagram represents the number of orthologous clusters that *T. cruzi* shares with five other species (see Fig. 2). The Venn diagram shows that there is only one gene cluster shared by all six species, indicating the conservation in lineage after speciation. We have not identified any clusters specific to *T. cruzi*, *T. cruzi marinkelli and T. congolense*. However, one cluster was identified specifc for *T. brucei gambiense* and *T. brucei brucei*, four clusters specific for *T. rangeli*. These represent in-paralog clusters suggesting lineage specific gene expansion in these gene families. The identified function of four clusters in *T. rangeli* is chromosome organization and nucleoside metabolic process. For *T. brucei brucei* cluster role is not annontated while for *T. brucei gambiense*, it is suggested to be involved in nucleotide metabolic process. We have identified one cluster of single copy genes in all six genomes, implying that even after divergence of species they have conserved the single copy status (see Supplementary Table 1). Considering only the species that do not cause any disease in humans, i.e. *T. brucei brucei*, *T. cruzi marinkelli*, and *T. rangeli*, we found a single cluster between *T. cruzi marinkelli* and *T. rangeli* having role in nitrogen compound metabolic process and cellular metabolic process. There were three clusters common between *T. brucei brucei & T. rangeli* with role in cell cycle and organization of organelles. Compartitive analysis of *T. cruzi* and *T. brucei gambiense* species identified one cluster conserved in Trypanosoma causing disease in humans and absent in other four species, directing towards novel pathogenic proteins specific to the human host.

### Domain Architecture of Ubiquitin Proteasome Pathway (UPP) proteins

**Figure 3:**
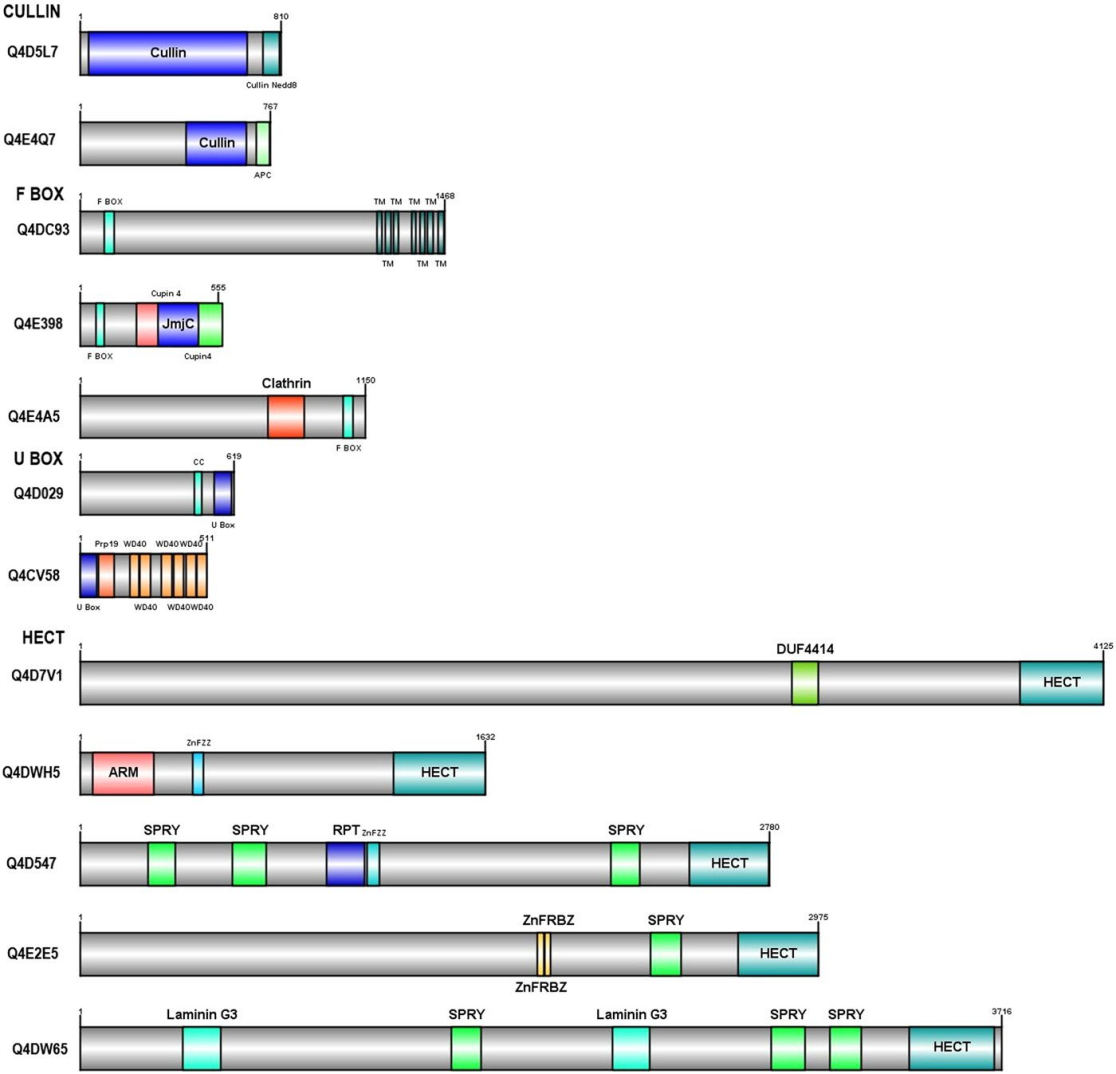
The Schematic Domain architectures of the RING E3 ligase proteins in *T. cruzi* are depicted. The full protein is colored in grey and other domain types are colored in different colors separately. In some of the domain architecture, two domains overlap, so they are represented by two names. The number at top depicts the length of each protein. Domain architecture of atypical UPP proteins based on the presence of unique appended domains or their extensions tethered at N- or C- terminus. Uniprot ids corresponding to each domain diagram is listed at the right. Abbreviations used: SP-signal Peptide, TM-Transmembrane domain, CC-coiled coil.

**Figure 4:**
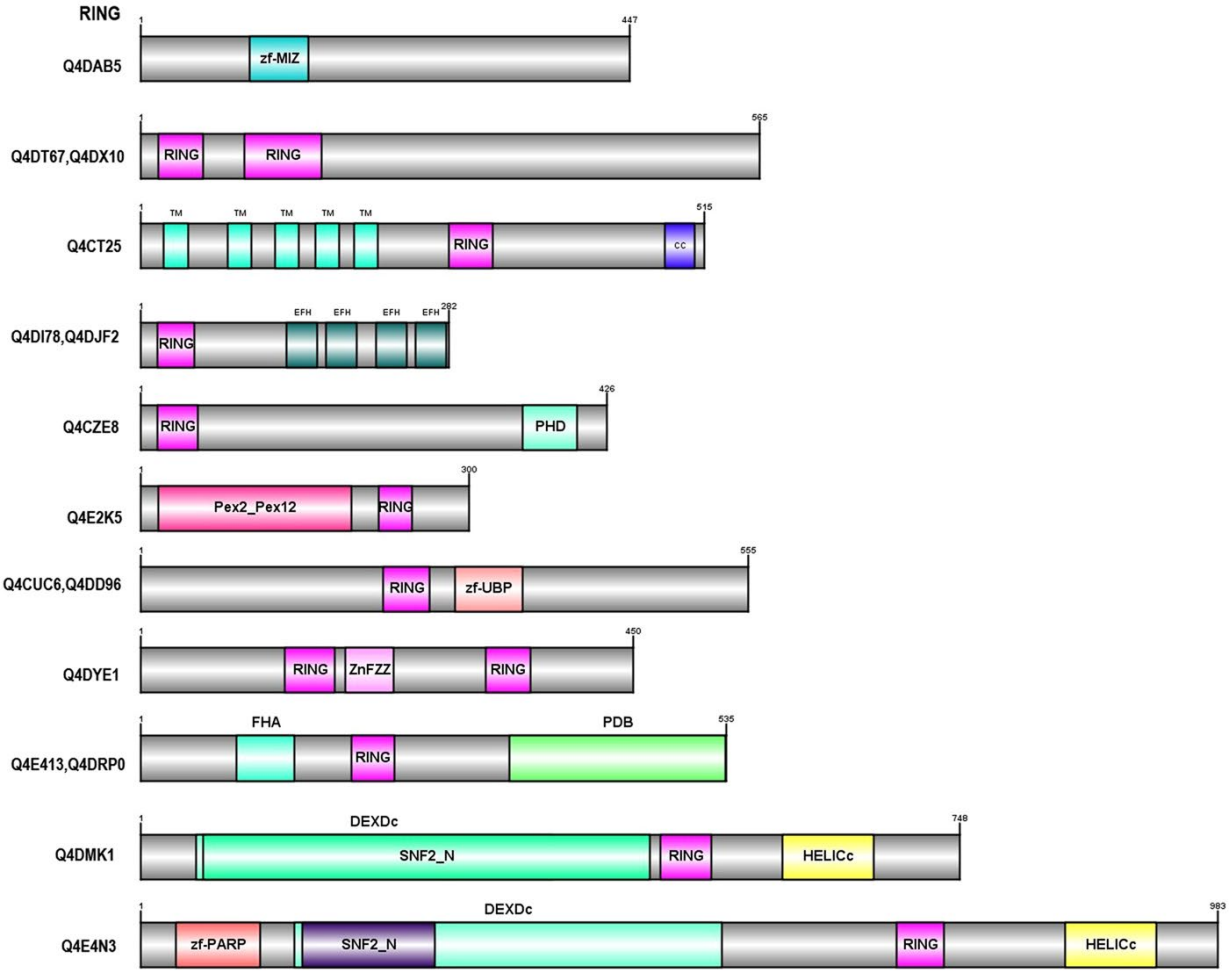
The Schematic Domain architectures of the Ubiquitin ligases (E3s) proteins in *T. cruzi* are depicted. The full protein backbone is colored in grey while other domain types are highlighted in different colors. In some of the domain architecture, two domains overlap, so they are represented by two names. The number at top depicts the length of each protein. Domain architecture of atypical UPP proteins based on the presence of unique appended domains or their extensions tethered at N- or C- terminus. Uniprot ids corresponding to each domain diagram is listed at the right. Abbreviations used: TM-Transmembrane domain, CC-coiled coil.

**Figure 5:**
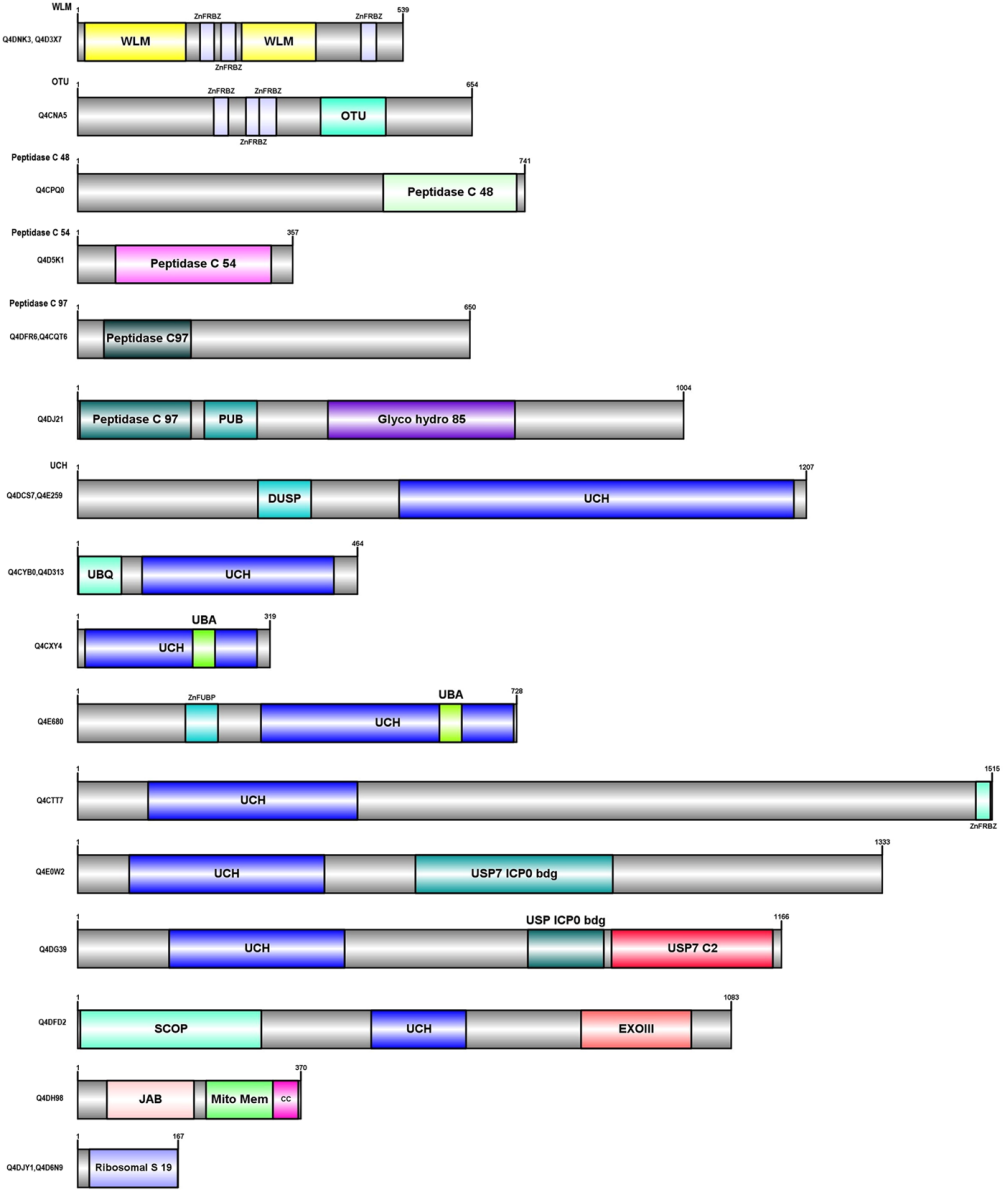
The Schematic Domain architectures of the Deubiquitinating enzymes (DUBs) proteins in *T. cruzi* are depicted. The full protein is colored in grey and other domain types are colored in different colors separately. In some of the domain architecture, two domains overlap, so they are represented by two names. The number at top depicts the length of each protein. Domain architecture of atypical UPP proteins based on the presence of unique appended domains or their extensions tethered at N- or C- terminus. Uniprot ids corresponding to each domain diagram is listed at the right. Abbreviations used: SP-signal Peptide, CC-coiled coil.

UPP proteins are multi-domain proteins typically consisting of a characteristic core domain or motif. In addition, some UPP proteins have additional functional domains that may be appended during biological evolution. Careful examination of E3s and DUBs predicted in *T. cruzi* using Pfam database showed that most of them have a generic modular architecture that adheres to a prototypical UPP. The functional relevance of the additional domains fused to typical UPP in *T. cruzi* needs to be experimentally explored. Clearly, the presence of unusual domain fusions in *T. cruzi* UPP suggests multiple functional roles for many of these *T. cruzi* UPP enzymes as has been shown in other organisms. We have also noticed the difference in the length of the E3s and DUBs suggesting another functional role that may be required for interacting with different types of proteins. We have schematically represented domain architecture of atypical UPP proteins based on the presence of unique appended domains or their extensions tethered at N- or C- terminus in Figs 3, 4 and 5. Below are the detailed descriptions of the UPP- E3s and DUBs.

### Ubiquitin/UBL ligases (E3s)

UPP proteins mainly consist of E3 ubiquitin/UBL ligases (~40.52%) and comprises very distinct cluster of proteins involved in specifically transferring Ub/Ubls to a given substrate. E3s are known for the substrate specificity and recognition and the presence of high percentage of E3s suggests the same. There are three superfamilies of E3 ubiquitin/UBL ligases: RING (Really Interesting New Gene) finger and U-box E3s, which are involved in multi-protein complexes whereas HECT ubiquitin ligases have a direct role in catalysis during ubiquitination. In Supplementary Table S2, we have summarized all potential E3 ubiquitin/UBL ligases that have been found in *T. cruzi*. We have discussed in detail various E3 ligases below:

#### RING domain ubiquitin ligases

RING is the largest family of E3 ligases and they mediate the transfer of E2 bound ubiquitin to substrates without thioester bond formation. They have RING domain, which is a cysteine/histidine rich zinc-binding domain and two zinc atoms are arranged in cross brace manner by cysteine/histidine residues^25^. In *T. cruzi*, about 67 putative RING E3s are identified and 60 of them have characteristic RING domain. Ring domain is present as one copy in most of the proteins but in some cases, it is present in two copies (see Fig. 3). Apart from RING, other predicted domains are DEXDc, HELICc, Pex2-Pex12, FHA, PHD, zf-B Box, zf-PARP, zf-UBP, EFH, PDB, ZnF_ZZ, SNF2_N, and zf-MIZ (see Supplementary Table S2).

#### Cullin E3 ligases

Cullin E3s contain evolutionary conserved cullin homology domain at N-terminus and interacts with RING protein at C-terminus. The specificity of Cullin-RING ubiquitin ligases (CRLs) is determined by substrate recognition receptors such as F-box proteins^26^. They organize the largest class of RING E3 ligase, known as cullin-RING ligase complexes. All cullin proteins have neddylation site except APC2 cullin. In *T. cruzi*, 13 cullin E3 ligases have been predicted, with a characterized cullin and cullin NEDD8 domain except for APC2 (Q4E4Q7), which has only the cullin domain (see Fig. 4). Cullin proteins are diverse in size ranging from 489 aa to 1230 aa. We have observed the presence of two cullin domains in 6 *T. cruzi* proteins (Q4CZ35, Q4DZU8, Q4CX33, Q4CTM0, Q4DVD1, Q4CUC0) with a distinct presence at the N- and C-terminus (see Supplementary Table S2).

#### F-BOX E3 ligases

The F-box domain in E3 ligases, is generally a component of SCF ubiquitin-ligase complexes. SCF complexes contain four components: Skp1, a cullin, Rbx1/Roc1/Hrt1, and an F-box protein that facilitates interaction between substrates and E2s^27^. The F-box proteins have the F-box motif at the N-terminus and it is coupled with other motifs of the SCF complex with the C-terminus using leucine rich and WD repeats. In *T. cruzi*, 8 putative F-box proteins (Q4CSG2, Q4DC93, Q4E398, Q4E4A5, Q4E3Q8, Q4CYB2, Q4DDI5, Q4D4D7) have been identified and interestingly 7 of them have F-box domain at N terminus while 1 of them have their presence at the C-terminus (see Fig. 4).

#### U-Box E3 ligases

There is a high structural similarity present between the U-box and the RING domain of E3 ligases but generally U-box E3s lacks the metal binding residues^5^. There is one sub-group of U-box proteins that is known to add a polyubiquitin chain on target protein resulting in formation of numerous branched structures with a distinct biological function. One of them is also known to act as a co-chaperone helps in regulating quality control of protein folding and with the help of ubiquitination activity, it degrades unfolded protein^28^. In *T.cruzi*, there are total 5 putative U-box proteins identified (Q4DW87, Q4D7U8, Q4D029, Q4DHT9, Q4CV58) (see Fig. 4). All the U-box E3s have this domain present at C-terminus except 1 U-box E3 (Q4CV58) where it is present at the N-terminus. We have also identified a homolog of CHIP (C-terminal of Hsp70-interacting protein) in *T. cruzi* (Q4DHT9) that is involved in protein quality control by ubiquitination of denatured proteins by using molecular chaperones Hsp90 and Hsc70^29^.

#### HECT domain E3 ligases

These category E3s are similar to C-terminus of E6-AP that is an E3 ligase. HECT E3s range from size 80 kDa to more than 500 kDa. The HECT domain is the catalytic domain and substrate specificity is determined by N-terminus extensions that can be WW domains, RCC1 like domains, ARM repeats and ZnF domain. In *T. cruzi*, total 16 putative HECT domain containing proteins are identified, with the characterized HECT domain ranging in different size and the protein length varying from 162 aa to 4423 aa (see Fig. 4). Other domains mainly identified are SPRY domain, ARM repeat, ZnF RBZ domain, ZnF ZZ domain, Laminin G3 domain, coiled coil and DUF 4414 (see Supplementary Table S2).

### Deubiquitinases (DUBs)

DUBs are the group of enzymes that can specifically cleave ubiquitin molecules that would help in rescue of proteins from degradation, recycle ubiquitin and play a role in controlling cellular signaling. The role of DUBs has also been illustrated in regulating membrane traffic, DNA repair pathways, transcriptional activity and protein quality control^30^. In *H. sapiens*, there are about more than 100 DUBs detailed so far^7^. In *T. cruzi*, we have identified about 72 putative DUBs and interestingly, a new class of WLM DUBs has also been observed that has not been characterized in *H. sapiens*. Although the presence of the WLM DUBs has been observed in *Plasmodium falciparum* and *Sacchromyces cereviscae*, in *T. cruzi*, we have identified 2 WLM DUBs (Q4D3X7, Q4DNK3) containing the WSS1-like metalloproteases domain that comes under Zn-dependent peptidase family-which is the catalytically active protease domain (see Fig. 5). UCH DUBs has the highest fraction (43.05%) compared to other DUBs in *T. cruzi* and in *H. sapiens* (58.57%). These proteins vary in length of 319 aa to 1515 aa and the size and position at N- or C-terminus of UCH domain is variable amongst them (see Supplementary Table S3).

### Localization of UPP machinery proteins

**Figure 6:**
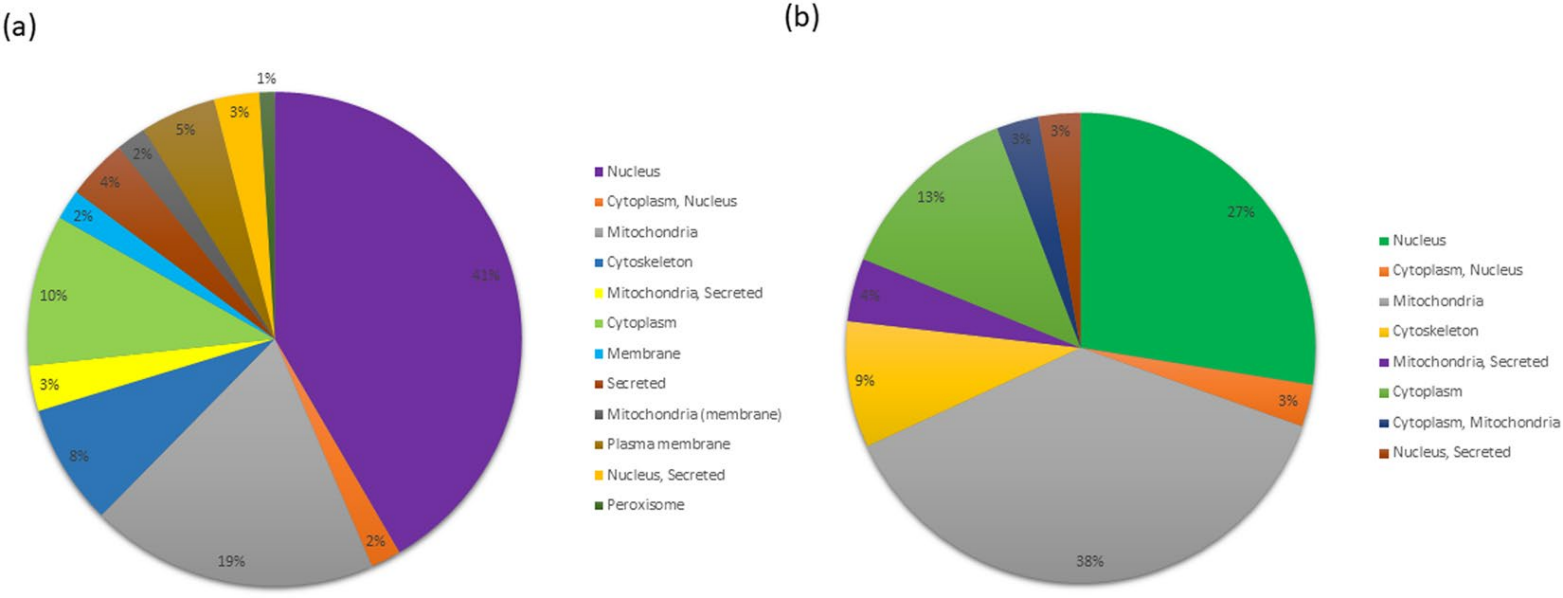
The pie-chart showing the percentage and cellular compartments of UPP proteins of *T. cruzi* using ProtSecKB database. (**a**) E3s localization. (**b**) DUBs localization. Each cellular compartment, with name and percentage of the total number of proteins of each class in each compartment, of the pie-chart is shown with distinct color.

The presence of multiple appended domains and diversified roles played by UPP proteins require their presence (transit) into various cellular compartments. We therefore analyzed *T. cruzi* UPP proteins for the presence of putative signal sequence for secretory protein and transit sequences for localization in mitochondria, nucleus, cytoskeleton, peroxisome etc. as predicted by ProtSeckB^31^. We have found that in 181 *T. cruzi* E3s and DUBs, the software was not able to predict localization of 11 proteins. Analysis of remaining proteins shows that 136 proteins are predicted to have a transit peptide and 15 signal peptides, possibly for directing them to different cellular organelles or as part of a secretory pathway. Some of the UPP proteins that are predicted to be present in the nucleus may cause protein degradation in the nucleus due to the presence of proteasome machinery in the nucleus in *Trypanosoma*, but in case of mitochondria, the proteins from the matrix or outer membrane of mitochondria are known to retro translocate to the cytoplasm for degradation. Some proteins present on the outer membrane of mitochondria may interact with their cytosolic domain (Q4CSG2, Q4DYQ0). E3s and DUBs are predicted to localize in multiple cellular organelles such as cytoplasm, nucleus, mitochondria, cytoskeleton, plasma membrane and peroxisome (see Fig. 6a and b). Localization of E3s and DUBs at different cell compartments indicates distinct substrates proteins ubiquitinated or deubiquitinated respectively (see Supplementary Table S4). The secreted proteins play a major role in invading and infecting humans and causing diseases. We have identified few secretory proteins in *T. cruzi* suggesting their role in host pathogenesis (see Supplementary Table S5). DUBs that are localized in mitochondria regulate mitochondrial morphology, while those localized in nucleus regulate nucleus structure and function. We have also predicted localization of few DUBs in more than one organelle. Such proteins would have more than one function and these could act as good drug targets to kill the pathogen. For example, our study has identified 2 putative DUBs (Q4CK98, Q4DPZ0) characterized by MPN and OTU domain respectively to be dually targeted to the cytoplasm and nucleus while other 2 putative DUBs (Q4D313, Q4CPQ0) having UCH and Peptidase C 48 domain respectively dually localized to cytoplasm and mitochondria. We managed to identify a homologue of DUB (Q4D313) as USP14 in *Homo sapiens* using blastp, which is a proteasome associated DUB and known for its role in neurodegenerative disease and cancer biology^32,33^. USP14 has been identified a good drug target for oncology and VLX1570 inhibitor is being designed and developed^34^. Experimental validation is required for the identified putative DUB (Q4D313) for its deubiquitinates activity. Futuristic studies of this DUB role in the proteasomal pathway of Trypanosoma may identify its role in pathogenesis and virulence. Similar inhibitors would be feasible to be used for antiparasitic drug therapy needs more detailed study. There are very limited numbers of studies on ubiquitin proteasome pathway proteins (E3 ligases and DUBS) in Trypanosoma parasite and identification of those proteins, which are particularly essential for the parasite survival or if the proteins are localized in different compartment as compare to human homologue are proposed to be a potential drug target.

### Expression analysis of T. cruzi UPP proteins

**Figure 7:**
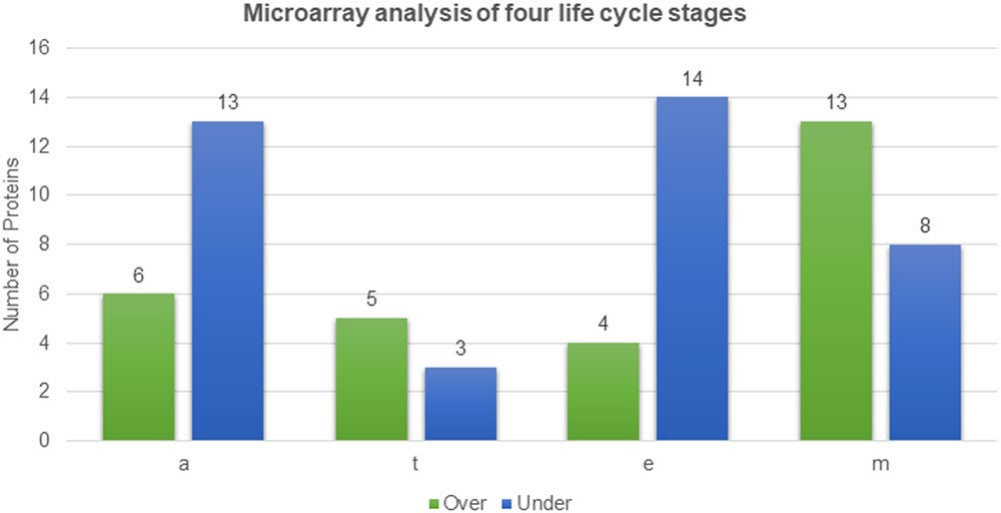
The UPP protein expression profile in four life cycle stages of *T. cruzi* based on microarray analysis data. The vertical axis shows the percentage of proteins over- or under-expressed in different life cycle stages and the horizontal axis shows the name of the life cycle stages. Abbreviations used: a-amastigote, t-trypomastigote, e-epimastigote, m-metacyclics.

To study the expression of UPP machinery proteins during life cycle stage of *T. cruzi*, transcriptomics data from Gene Expressions Omnibus repository was used. In *T. cruzi*, there are four different life cycle stages, with transmission between its two hosts- the human and in triatomine bug. In human, the *T. cruzi* stages are amastigotes and trypomastigotes while epimastigotes and metacyclics occur in the triatomine bug. To avoid false negatives, we used a lenient cutoff value of ±1.2 to find over- and under-expressed proteins at a stage. Though this cutoff has some chance of false positives, the objective was to maximize sensitivity rather than specificity. Of all the differentially expressed UPP proteins, we found most of those to be overexpressed in metacyclics and under-expressed in amastigotes (see Fig. 7). The 6 over-expressed UPP proteins in the amastigote stage (see Supplementary Table S6) suggest that these have an important role in controlling the *Trypanosoma* pathogenesis when the parasite shifts from the trypomastigote to the amastigote stage. This may be a viable pathogen strategy to control its survival and pathogenesis. Further studies on the individual proteins and the pathways involved, can be a new strategy in designing and developing better and suitable drugs against the parasite.

### Ubiquitin Proteasome Pathway as a drug target

Ubiquitin proteasome is a major pathway for the degradation of the intracellular proteins but also plays a role in other cellular processes including cell cycle progression, differentiation, angiogenesis, immune response, viral response and apoptosis^11,35,36^. Dysfunction of ubiquitin proteasome system leads to malignancies, disorders of immune response, neurodegenerative diseases, muscle wasting and genetic diseases like cystic fibrosis, Angelman’s and Liddle syndrome^8,11,37^. Drugs targeting UPP components usually are proteasome inhibitors or drugs specifically binding to E3s or to substrates and inhibitors for DUBs. Recently, UPP has gained a lot of momentum as a drug targeting machinery in parasites too. In apicomplexan parasites such as *P. falciparum*, E3 ligase HRD1 is suggested to be a good drug target due to very poor sequence similarity with its host homologue^38^. Similarly a malarial UCHL3 DUB is shown to be essential for the parasite survival and structural studies have identified distinct ubiquitin binding site in PfUCHL3 in comparison to its host human suggesting it to be a potential drug target^39^. In *Leishmania*, Ufm1 overexpression results in reduced survival of parasite in the human macrophage, implying it could serve as a suitable drug target^40^. The proteasome subunits of *T. cruzi* have been studied and it has shown that the proteasome inhibitors lactacystin and MG132 inhibit the transformation of trypomastigotes to amastigotes^21^. Another inhibitor, Bortezomib, which is commonly used to treat myeloma, is also shown to inhibit stages of *Trypansosoma*^41^. While the proteasome potential for drug targets has been indicated, the components of UPP pathway have not been characterized and studied. We, therefore highlighted and discussed the UPP pathways components here, given the importance in a wide variety of processes, could act as potential drug targets for treating *Trypanosoma* diseases. However, the importance of UPP proteins as a drug target in other parasites, have already been studied and now are used to treat the parasitic diseases.

From our study, we have identified few proteins in *T. cruzi* with a distinct domain that are absent in its host (human), which we propose to be explored as a potential drug target. There are F-box E3 ligases (Q4E398, Q4E4A5) that have the JmjC, Cupin4 and Clathrin domain are not found in *H. sapiens* when performed the similarity search. Similarly, a new class of DUBs, WLM is predicted in *T. cruzi* (Q4D3X7, Q4DNK3), which is absent in *H. sapiens* serves as a potential new drug target. The other examples are Peptidase C97 DUBs (Q4DM33, Q4DJ21), which has the PUB domain and Glyco hydrolases domain that are also absent in the host. It has been reported for the cytoskeleton proteins, ubiquitin-dependent degradation occurs when the parasite transforms from metacyclic trypomastigote to amastigote. Therefore, this step can be targeted where degradation of the cytoskeleton proteins can be inhibited and therefore, can hamper the survival of the parasite inside the host by blocking its entry inside host. Identification and experimental characterization of components involved in degradation pathway and their regulatory proteins will help in developing drugs for these neglected diseases. Relevant to this we have also identified five E3 ligases (Q4D5L7, Q4D6X2, Q4D7U8, Q4D897, Q4DSZ1) and a DUB (Q4DMB6) in the amastigote stage. These proteins present only in amastigote stage suggest it to be potential drug targets, which may prevent the transmission of the parasite from amastigote stage to trypomastigote stage. In humans there are many E3 ligases and DUBs are being explored as drug targets for cancer, neurodegenerative, metabolic disease and presence of different catalytic mechanism in E3 ligases give them better specificity, suggesting them to be potential drug target in Trypanosoma. Studies of these proteins in pathogenesis of Trypanosoma and how different these proteins are from their human homologues would really help in design of the inhibitors with minimum toxicity and more specificity.

## Conclusion

Ubiquitin proteasome pathway components are the ubiquitous enzymes essential for protein turnover and dictate the half-life of the proteins. This study has identified 269 proteins in *T. cruzi*, involved putatively in UPP. We have carried out comparative genomic analysis in order to identify orthologous UPP clusters among the different species of Trypanosoma. We have characterized *T. cruzi* UPP components extensively in terms of their domain architecture, cellular localization and protein expression profiles. Based on our *in-silico* analyses, we have identified novel class of WLM DUBs in *T. cruzi* and predicted several distinct E3s and DUBs where the homologue of the same is absent in human host, suggesting these proteins to be a potential drug target. We have also identified majority of the peculiar RING E3 ligases suggests interaction with various target specific substrates and regulate numerous cellular processes, as in other organisms. Our blastp search helped in predicting few homologues of *T. cruzi* UPP proteins in humans. The presence of the same domain in *T. cruzi* indicates the similar function in parasite too but needs to be experimentally explored. Interestingly, there were many *T. cruzi* UPP proteins for which the homologues are absent in human, suggesting their role in other diverse function of the parasite that could be linked to the survival and pathogenicity of the parasite. Our localization prediction analysis revealed the presence of some proteins in two different compartments of the cell, implying their role in regulating diverse cellular functions in cellular space. One of the *T. cruzi* E3 ligase (SPRING) has been reported to be secreted in the host cytoplasm, utilize host E2s, E1s to degrade host proteins and promote parasite survival^42^. Our analyses also revealed many other UPP proteins with a putative signal peptide to get secreted and could modify host cellular function and we posit it could act as a virulence factor. Overall, characterization of the UPP proteins will help in understanding biological process of the parasite and would help in developing new drugs. Microarray expression data predicted the presence of UPP proteins in all four stages of parasite and also help in distinguishing proteins expressed specifically in amastigote stage and these would help in intracellular endurance of pathogen inside host. This study would assist in the delineation of the ubiquitin proteasome pathway in *Trypanosoma* and provide support for experimental studies on this pathway.

## Methods

### Protein and Pfam domain datasets for UPP

Full proteome FASTA databases from the following organism were downloaded from Uniprot:- *T. cruzi* (19242 sequences, Proteome Id: UP000002296), *T. brucei gambisense* (9668 sequences, Proteome Id: UP000002316), *T. brucei brucei* (8587 sequences, Proteome Id: UP000008524), *T. cruzi marinkelli* (10052 sequences, Proteome Id: UP000007350), *T. congolense* (5906 sequences, Proteome Id: UP000000702), *T. rangeli* (7365 sequences, Proteome Id: UP000031737), *T. vivax* (3778 sequences, Proteome Id: UP000009027), and *Homo sapiens* (70946 sequences, Proteome Id: UP000005640)^43^. Twenty-four Pfam motifs found in ubiquitin proteasome system were selected and downloaded from Pfam HMM library version 22.0^44^. The following Pfam domains were included Ufm1, PF03671; APG12, PF04110; Atg8, PF02991; ubiquitin, PF00240; Urm1, PF09138; ThiF, PF00899; UBACT, PF02134; UQ-con, PF00179; zf-C3HC4, PF00097; zf-RING-like, PF08746; zf-MIZ, PF02891; HECT, PF00632; Cullin, PF00888; U-box, PF04564; F-box, PF00646; OTU, PF02338; Josephin, PF02099; JAB, PF01398; DUF862, PF05903; WLM, PF08325; UCH, PF00443; Peptidase C12, PF01088; Peptidase C48, PF02902; and Peptidase C54, PF03416^23,24^.

### Domain similarity searches for protein identification

HMMER Version 3.1b1, downloaded from http://hmmer.org, was used in the study^45^. The Pfam profiles were searched against all downloaded FASTA protein databases using hmmsearch program, which was automated using a perl script. HMM searches were performed using different threshold E-values (from 0.1, 0.2, and so on, up to 1.0) to evaluate sensitivity of results at different thresholds. Since all the threshold values had identical output, the output from threshold of 0.1 E-value was selected for all subsequent analysis.

### Comparative Genomic analysis

The genome-wide comparison and annotation of clusters of orthologous groups were generated by the web server OrthoVenn (http://aegilops.wheat.ucdavis.edu/OrthoVenn)^46^. As input, OrthoVenn was provided with the FASTA sequence of all species. The default e-value cut-off of 1e-5 and inflation value (−I) of 1.5 was used to perform orthologous cluster analysis. Since OrthoVenn can only take six species at a time, we performed the analysis in two batches, keeping *T. cruzi* as base. We then parsed the results with in house Perl scripts to map the list of proteins orthologs to the 269 UPPs we found in *T. cruzi*. We also searched OrthoVenn with the FASTA sequences retrieved for UPP proteins from *T. cruzi* (Tc)*, T. brucei gambiense* (Tbg), *T. brucei brucei* (Tbb), *T. cruzi marinkelli* (Tcm), *T. congolense* (Tcg), *T. rangeli* (Tr) resulting from hmmsearch. The search in OrthoVenn was limited to six species and number of identified UPP proteins were just 12 in T. *vivax (*Tv*)* so it was omitted from our search query. In the comparative analysis of putative UPPs amongst the species, only six species were selected for OrthoVenn analysis.

### Functional Domain architecture search

The HMM results for all species were parsed to filter matched proteins and the respective FASTA sequences were fetched using in house perl scripts. These proteins found through hmmer searches are homologs of all the E3s and DUBs according to hmmer results. The resulting FASTA files were searched for their functional domains using the batch access of SMART database^47^ at http://smart.embl-heidelberg.de/smart/batch.pl. The results from *T. cruzi* were analyzed further in detail. Protein domain architecture schematics were made with Illustrator of Biological Sequences(IBS) software^48^.

### Subcellular Localization prediction

The proteins filtered from HMMER results were searched in ProtSecKB database^31^ at http://bioinformatics.ysu.edu/secretomes/protist/index.php for known and predicted subcellular localizations. The results for UPP enzymes were analyzed to study whether they were found in single or multiple-subcellular localizations.

### Expression analysis

To analyze gene expression profiles of the UPP machinery proteins in *T. cruzi*, the whole genome oligonucleotide microarray data from Minning *et al*.^49^ study was used. The data was downloaded from GEO and parsed using in house perl scripts. Genes with missing values were removed and remaining data analyzed further for expression patterns.

### Data availability

The datasets supporting the results of the article are included within this published article and its additional files.

## Electronic supplementary material

Supplementary Table S2-6

Supplementary Table S1

## References

[1] Hochstrasser M (2000). Evolution and function of ubiquitin-like protein-conjugation systems. Nature cell biology.

[2] Kerscher O, Felberbaum R, Hochstrasser M (2006). Modification of proteins by ubiquitin and ubiquitin-like proteins. Annu. Rev. Cell Dev. Biol..

[3] Huibregtse JM, Scheffner M, Beaudenon S, Howley PM (1995). A family of proteins structurally and functionally related to the E6-AP ubiquitin-protein ligase. Proceedings of the National Academy of Sciences.

[4] Budhidarmo R, Nakatani Y, Day CL (2012). RINGs hold the key to ubiquitin transfer. Trends in biochemical sciences.

[5] Hatakeyama S, Yada M, Matsumoto M, Ishida N, Nakayama K-I (2001). U box proteins as a new family of ubiquitin-protein ligases. Journal of Biological Chemistry.

[6] Saurin AJ, Borden KL, Boddy MN, Freemont PS (1996). Does this have a familiar RING?. Trends in biochemical sciences.

[7] Nijman SM (2005). A genomic and functional inventory of deubiquitinating enzymes. Cell.

[8] Powell SR, Herrmann J, Lerman A, Patterson C, Wang X (2012). The ubiquitin–proteasome system and cardiovascular disease. Progress in molecular biology and translational science.

[9] Dantuma NP, Bott LC (2014). The ubiquitin-proteasome system in neurodegenerative diseases: precipitating factor, yet part of the solution. Frontiers in molecular neuroscience.

[10] Frezza M, Schmitt S, Ping Dou Q (2011). Targeting the ubiquitin-proteasome pathway: an emerging concept in cancer therapy. Current topics in medicinal chemistry.

[11] Wang J, Maldonado MA (2006). The ubiquitin-proteasome system and its role in inflammatory and autoimmune diseases. Cell Mol Immunol.

[12] Kirchhoff LV (2011). 1 Epidemiology of American Trypanosomiasis (Chagas Disease). Advances in parasitology.

[13] Tyler K, Engman D (2001). The life cycle of Trypanosoma cruzi revisited. International journal for parasitology.

[14] Beaumier CM (2016). Status of vaccine research and development of vaccines for Chagas disease. Vaccine.

[15] Dias JCP, Coura JR, Yasuda MAS (2014). The present situation, challenges, and perspectives regarding the production and utilization of effective drugs against human Chagas disease. Revista da Sociedade Brasileira de Medicina Tropical.

[16] Kirchhoff L, Kim KS, Engman D, Donelson JE (1988). Ubiquitin genes in trypanosomatidae. Journal of Biological Chemistry.

[17] Télles S, Abate T, Slezynger TC, Henríquez DA (1999). Trypanosoma cruzi and human ubiquitin are immunologically distinct proteins despite only three amino acid difference in their primary sequence. FEMS Immunology & Medical Microbiology.

[18] de Diego JL (2001). The Ubiquitin− Proteasome Pathway Plays an Essential Role in Proteolysis during Trypanosoma cruzi Remodeling. Biochemistry.

[19] Kurup SP, Tarleton RL (2014). The Trypanosoma cruzi flagellum is discarded via asymmetric cell division following invasion and provides early targets for protective CD8+ T cells. Cell host & microbe.

[20] Gonzalez J (1996). Proteasome activity is required for the stage-specific transformation of a protozoan parasite. Journal of Experimental Medicine.

[21] Cardoso J (2011). Analysis of proteasomal proteolysis during the *in vitro* metacyclogenesis of Trypanosoma cruzi. PloS one.

[22] Muñoz, C., San Francisco, J., Gutiérrez, B. & González, J. Role of the ubiquitin-proteasome systems in the biology and virulence of protozoan parasites. *BioMed research international* **2015** (2015). 10.1155/2015/141526 PMC4452248 26090380

[23] Choy A (2013). Decoding the ubiquitin-mediated pathway of arthropod disease vectors. PloS one.

[24] Ponts N (2008). Deciphering the ubiquitin-mediated pathway in apicomplexan parasites: a potential strategy to interfere with parasite virulence. PloS one.

[25] Metzger MB, Pruneda JN, Klevit RE, Weissman AM (2014). RING-type E3 ligases: master manipulators of E2 ubiquitin-conjugating enzymes and ubiquitination. Biochimica et Biophysica Acta (BBA)-Molecular Cell Research.

[26] Bulatov E, Ciulli A (2015). Targeting Cullin–RING E3 ubiquitin ligases for drug discovery: structure, assembly and small-molecule modulation. Biochemical Journal.

[27] Zheng N (2002). Structure of the Cul1–Rbx1–Skp1–F boxSkp2 SCF ubiquitin ligase complex. Nature.

[28] Hatakeyama S, Matsumoto M, Yada M, Nakayama KI (2004). Interaction of U‐box‐type ubiquitin‐protein ligases (E3s) with molecular chaperones. Genes to Cells.

[29] Murata S, Chiba T, Tanaka K (2003). CHIP: a quality-control E3 ligase collaborating with molecular chaperones. The international journal of biochemistry & cell biology.

[30] Clague MJ, Coulson JM, Urbé S (2012). Cellular functions of the DUBs. J Cell Sci.

[31] Powell, B. *et al*. ProtSecKB: The Protist Secretome and Subcellular Proteome Knowledgebase. *Computational Molecular Biology* **6** (2016).

[32] Zhang B, Li M, Huang P, Guan XY, Zhu YH (2017). Overexpression of ubiquitin specific peptidase 14 predicts unfavorable prognosis in esophageal squamous cell carcinoma. Thoracic cancer.

[33] Chen P-C (2009). The proteasome-associated deubiquitinating enzyme Usp14 is essential for the maintenance of synaptic ubiquitin levels and the development of neuromuscular junctions. Journal of Neuroscience.

[34] Wang X (2016). The proteasome deubiquitinase inhibitor VLX1570 shows selectivity for ubiquitin-specific protease-14 and induces apoptosis of multiple myeloma cells. Scientific Reports.

[35] McBride WH, Iwamoto KS, Syljuasen R, Pervan M, Pajonk F (2003). The role of the ubiquitin/proteasome system in cellular responses to radiation. Oncogene.

[36] Rahimi N (2012). The ubiquitin-proteasome system meets angiogenesis. Molecular cancer therapeutics.

[37] Tomaić V, Banks L (2015). Angelman syndrome-associated ubiquitin ligase UBE3A/E6AP mutants interfere with the proteolytic activity of the proteasome. Cell death & disease.

[38] Chung D-WD, Ponts N, Prudhomme J, Rodrigues EM, Le Roch KG (2012). Characterization of the ubiquitylating components of the human malaria parasite’s protein degradation pathway. PloS one.

[39] Artavanis-Tsakonas K (2010). Characterization and structural studies of the Plasmodium falciparum ubiquitin and Nedd8 hydrolase UCHL3. Journal of Biological Chemistry.

[40] Gannavaram S, Davey S, Lakhal-Naouar I, Duncan R, Nakhasi HL (2014). Deletion of ubiquitin fold modifier protein Ufm1 processing peptidase Ufsp in L. donovani abolishes Ufm1 processing and alters pathogenesis. PLoS neglected tropical diseases.

[41] Steverding D, Wang X (2009). Trypanocidal activity of the proteasome inhibitor and anti-cancer drug bortezomib. Parasites & vectors.

[42] Hashimoto M, Murata E, Aoki T (2010). Secretory protein with RING finger domain (SPRING) specific to Trypanosoma cruzi is directed, as a ubiquitin ligase related protein, to the nucleus of host cells. Cellular microbiology.

[43] Consortium U (2017). UniProt: the universal protein knowledgebase. Nucleic acids research.

[44] Finn RD (2006). Pfam: clans, web tools and services. Nucleic acids research.

[45] Finn RD, Clements J, Eddy SR (2011). HMMER web server: interactive sequence similarity searching. Nucleic acids research.

[46] Wang Y, Coleman-Derr D, Chen G, Gu YQ (2015). OrthoVenn: a web server for genome wide comparison and annotation of orthologous clusters across multiple species. Nucleic acids research.

[47] Letunic I, Doerks T, Bork P (2014). SMART: recent updates, new developments and status in 2015. Nucleic acids research.

[48] Liu W (2015). IBS: an illustrator for the presentation and visualization of biological sequences. Bioinformatics.

[49] Minning TA, Weatherly DB, Atwood J, Orlando R, Tarleton RL (2009). The steady-state transcriptome of the four major life-cycle stages of Trypanosoma cruzi. BMC genomics.

