# Supplementary material for: Ubiquitin Proteasome pathway proteins as potential drug targets in parasite Trypanosoma cruzi
Source: Sci Rep. 2018 May 30;8:8399. doi: 10.1038/s41598-018-26532-z (PMC5976635; doi:10.1038/s41598-018-26532-z)
Supplement: Supplementary file 1 — Supplementary Table S2-6 [file 41598_2018_26532_MOESM1_ESM.pdf]

Ubiquitin Proteasome pathway as potential drug target in parasite Trypanosoma cruzi

Ishita Gupta[1,2], Suruchi Aggarwal,[2] Kanika Singh[2], Amit Yadav[2], Sameena Khan*[2]

Address:[1] Structural Immunology Group, International Centre for Genetic Engineering and Biotechnology, New Delhi 110067, India.[2] Drug Discovery Research Centre, Translational Health Science and Technology Institute, Faridabad 121001, India.

Email: Ishita Gupta- ishita@icgeb.res.in; Suruchi Aggarwal-suruchi@thsti.res.in; Kanika Singh-ksingh@thsti.res.in; Amit Kumar Yadav-amit.yadav@thsti.res.in; Sameena Khan*- sameena@thsti.res.in

*Corresponding author

Supplementary Table S1: **Orthologous clusters predicted by OrthoVenn for species**

It is included as excel file.

Supplementary Table S2: **Predicted Number of domains of E3s in *T. cruzi***

**E3s classification**	**Domain**	**Uniprot IDS ***	**Predicted Functions**
RING	RING	Q4DYQ0,Q4D1I0,Q4DB31,Q4DI78,Q4DJF2,Q4CKL0,Q4DAL0,Q4E2K5,Q4E1T1,Q4DL73,Q4DPJ5,Q4DT67,Q4DXI0,Q4D0X0,Q4CZE8,Q4CY74,Q4DSB2,Q4CR46,Q4CVD0,Q4DMK1,Q4CKK6,Q4CKV4,Q4DP57,Q4E413,Q4CS38,Q4DKZ7,Q4DW46,Q4DZ28,Q4DRP0,Q4E5S0,Q4D8Z5,Q4DDL9,Q4CUC6,Q4DD96,Q4D662,Q4DR65,Q4E4N3,Q4DIY7,Q4D6F3,Q4D072,Q4CQH2,Q4D6X2,Q4CT25,Q4E0X7,Q4E1P7,Q4D2A5,Q4D0Q6,Q4D821,Q4DYE1,Q4DLU8,Q4DXD8,Q4DAZ0,Q4CPI2,Q4D328,Q4CPT2,Q4DCF4,Q4E3Z8,Q4D897,Q4DNX6, Q4E4Q1	Plays a role in protein-protein and protein-DNA interactions
RING	DEXDc	Q4DMK1, Q4E4N3	DNA damage repair
RING	HELICc	Q4DMK1, Q4E4N3	Helicase activity
RING	FHA	Q4E413, Q4DRP0	Recruited to E2 or their substrates by phosphorylation dependent protein-protein interactions and plays a role in initiation of cell cycle arrest at G2/M phase [1]
RING	PHD	Q4CZE8, Q4CY74, Q4E4N3, Q4DIY7, Q4D6F3	May bind to E2 ubiquitin ligase [2]
RING	Pex2_Pex12	Q4E2K5	Role in peroxisomal biogenesis by mediating polyubiquitination and monoubiquitination of Pex5 and also plays role in receptor recycling [3]
RING	zf-B_box	Q4DT67, Q4DXI0	Have a zinc finger that helps in protein-protein interactions
RING	zf-PARP	Q4E4N3	Regulatory component in DNA damage, by poly ADP-ribosylation of many target proteins such as core

			histones, linker histones H1 and transcription factors [4]
RING	PDB	Q4DRP0, Q4E413	Protein-protein interaction
RING	SNF2_N	Q4DMK1, Q4E4N3	ATPase of the SNF2/SWI multi-subunit complex
RING	EFH	Q4DI78, Q4DJF2, Q4D662, Q4DR65	Calcium-binding domain
RING	ZnF_ZZ	Q4DYE1	Mediating protein-protein interactions
RING	zf-UBP	Q4DD96, Q4CUC6	Binds to monoubiquitin and polyubiquitin chains
RING	Signal Peptide	Q4D897	Protein transferred to secretory pathway
RING	zf-MIZ	Q4D897, Q4DNX6, Q4E4T0, Q4CSC6, Q4DAB5, Q4D4T1, Q4CSJ0, Q4E655	Acts as a SUMO E3 ligase
RING	Transmembrane domain	Q4DYQ0, Q4E2K5, Q4E1T1, Q4CR46, Q4DIY7, Q4CT25, Q4E0X7, Q4D328, Q4CPT2, Q4E4Q1	Helps to anchor proteins to membrane
RING	C3H1	Q4DYL1, Q4DCC7	Play a role in RNA binding
Cullin	Cullin	Q4CZ35, Q4DGF2, Q4DZU8, Q4CX33, Q4CTM0, Q4D5L7, Q4DVD1, Q4CUC0, Q4D018, Q4CRP5, Q4DB05, Q4CX65, Q4E4Q7	Serve as a scaffold to bind to an adaptor protein at N-terminus and interacts with RING protein at C-terminus
Cullin	Cullin NEDD8	Q4CZ35, Q4DGF2, Q4DZU8, Q4CX33, Q4CTM0, Q4D5L7, Q4DVD1, Q4CUC0, Q4D018, Q4CRP5, Q4DB05, Q4CX65	Play role in Neddylation results in covalent conjugation of a NEDD8 moiety onto a conserved cullin lysine residue
Cullin	APC	Q4E4Q7	Controls the M/G1 transition through ubiquitination of cyclins and other mitotic effectors.
F Box	FBOX	Q4CSG2, Q4DC93, Q4E398, Q4E4A5, Q4E3Q8, Q4CYB2, Q4DDI5, Q4D4D7	Site of protein-protein interaction
F Box	JmjC	Q4E398	Histone demethylation and as protein hydroxylases [5]
F Box	Cupin_4	Q4E398	Role not established
F Box	Cupin_8	Q4E398	Role not established

F Box	Clathrin	Q4E4A5	Peculiar CHCR repeat that plays a role in protein-protein interaction and clathrin binding
F box	Transmembrane domain	Q4CSG2, Q4DC93, Q4D4J1	Helps to anchor proteins to membrane
U Box	Ubox	Q4DW87, Q4D7U8, Q4D029, Q4DHT9, Q4CV58	Known to carry the ubiquitination process in the absence of other E3 components
U Box	Prp19	Q4CV58	Plays a role in regulating spliceosome activity by targeting proteins for ubiquitination [6]
U Box	WD40	Q4CV58	Helping in recruitment of other proteins for protein-protein interactions
U Box	Coiled coil	Q4D029, Q4DHT9, Q4CV58	Diverse biological functions
HECT	HECT	Q4D7V1, Q4DG19, Q4E0S6, Q4DWH5, Q4DLE5, Q4DZM6, Q4DW65, Q4DSH5, Q4DX31, Q4D5C1, Q4DWV9, Q4E2E5, Q4D547, Q4CK94, Q4DSZ1, Q4CQA0	Catalysis reaction, Ub bound to E2 is transferred first to E3 cysteine and then transferred to lysine residue of substrate
HECT	ZnF_ZZ	Q4DWH5, Q4D547	Plays role in protein-protein interaction
HECT	SPRY	Q4DSH5, Q4DW65, Q4D547, Q4E2E5	Recognition of ubiquitinated substrate
HECT	ZnF_RBZ	Q4E2E5	Ubiquitin binding function
HECT	Laminin_G_3	Q4DW65	Target HECT to sugar binding molecules
HECT	Coiled coil	Q4D7V1, Q4E0S6, Q4CKK6, Q4DP57, Q4CS38, Q4E5S0, Q4D8Z5, Q4DDL9, Q4CT25, Q4E0X7, Q4DXD8, Q4DAZ0	Diverse biological functions
HECT	ARM	Q4DWH5	Identify the ubiquitination degradation signals in substrates
HECT	DUF4414	Q4D7V1	Binding function is not known but is found in DNA binding ubiquitin ligases

Domain name abbreviations used are *:* zfMIZ- Zinc finger Msx-interacting-zinc finger, RING- Really interesting new gene finger, EFH- Calcium binding domain, PHD- Plant homeodomain finger, Zf-UBP- Zn-finger in ubiquitin-hydrolases, ZNFZZ-, FHA- Forkhead associated domain ,DEXDc- DEAD-like helicases, HELICc- Helicase superfamily c-terminal domain, Zf-PARP- Zn-finger region Poly(ADP ribose) polymerase, Cullin NEDD8-Cullin protein neddylation domain, APC- Anaphase Promoting complex , TM- Transmembrane, JmjC- Jumonji Cupin family,Prp19-Pre-mRNA-processing factor 19,Wd40- Beta-transducin repeats, HECT- Homologous to the E6-AP Carboxyl Terminus ,DUF4414- Domain of unknown function 4414 , ARM- , RPT- Internal repeats, SPRY- SPla and the Ryanodine Receptor, ZnFRBZ- Zinc finger domain in Ran-binding proteins ,ZNFZZ- Zinc-binding domain.

** Some of the UniProt ids are present in more than one domain category as they are comprised of multiple domains.*

Supplementary Table S3: **Predicted Number of domains of DUBs in *T. cruzi.***

**DUBs Classification**	**Domains**	**Uniprot Ids***	**Predicted Functions**
WLM	WLM	Q4D3X7, Q4DNK3	Desumoylation
WLM	ZnF RBZ	Q4D3X7, Q4DNK3	Ubiquitin binding
WLM	zf-RanBP	Q4D3X7, Q4DNK3	Ubiquitin binding
OTU	OTU	Q4CNA5, Q4DPR2, Q4D5L1, Q4D0P2, Q4DFK1, Q4DNJ4, Q4DPZ0, Q4DPS6	Comprises the proteolytic catalytic triad
OTU	ZnF RBZ	Q4CNA5, Q4DPR2	Ubiquitin binding
JAB	JAB	Q4DKG4, Q4CYA0, Q4DPM7, Q4DDT1, Q4DH98, Q4CYA6, Q4DL11, Q4CUS2, Q4D3R6, Q4CMJ7, Q4CK98, Q4DG61, Q4DXR1, Q4DQZ2	Target the K63-linked polyubiquitin chains [7]
JAB	MitMem	Q4DH98, Q4CYA6	Normal Mitochondria morphology and function
JAB	Coiled coil	Q4DH98, Q4CYA6, Q4CUS2, Q4D3R6, Q4CK98, Q4CMJ7	Diverse biological functions
JAB	Signal peptide	Q4CYA0, Q4DXR1, Q4DQZ2	Protein transferred to secretory pathway
Peptidase C48	Peptidase C48	Q4DDC1, Q4CPQ0, Q4E4I8	Maturation of SUMO proteins (C-terminal hydrolase activity) and removal of SUMO from targets (isopeptidase activity)
Peptidase C54	Peptidase C54	Q4D5K1, Q4DFC9	Associated with Urm1 proteases

Peptidase C97	Peptidase C97	Q4DJ21, Q4DM33, Q4E565, Q4DWL2, Q4DFR6, Q4CQT6, Q4E560, Q4DWK7, Q4D3X5, Q4DNK1	Thiol peptidase activity
Peptidase C97	PUB	Q4DJ21, Q4DM33	Associated with ubiquitin
Peptidase C97	Glyco hydro 85	Q4DJ21, Q4DM33	Function not established
UCH	UCH	Q4DCS7, Q4E259, Q4D6N2, Q4E391, Q4DG39, Q4E680, Q4DYG8, Q4DS35, Q4CRZ3, Q4CYB0, Q4D313, Q4CTT7, Q4DFS1, Q4DMB6, Q4E0W2, Q4DTJ5, Q4DRN4, Q4E407, Q4D1W6, Q4DNK8, Q4CTT9, Q4E0K4, Q4DW10, Q4CXY4, Q4DF80, Q4D0S8, Q4CL15, Q4DFD2, Q4CUI7	Hydrolyze small amides and esters at the C-terminus of ubiquitin
UCH	DUSP	Q4DCS7, Q4E259	Role in ubiquitin specific protease activity
UCH	USP7 C2	Q4DG39	Role not established
UCH	USP7 ICP0 bdg	Q4DG39, Q4E0W2	Interact with herpesvirus 1 trans-acting transcriptional protein E3 ligase ICP0/VMW110 [8]
UCH	zf UBP	Q4E680	Functions as isopeptidase by cleaving isopeptide bond between ubiquitin moieties
UCH	UBA	Q4E680, Q4CXY4	Interaction of ubiquitin
UCH	ZnFRBZ	Q4CRZ3, Q4CL15	Ubiquitin binding function
UCH	UBQ	Q4CYB0, Q4D313, Q4CL15	Role in ubiquitin hydrolyase activity
UCH	SCOP	Q4DFD2	Plays role in protein-protein interaction
UCH	EXOIII	Q4DFD2	Stimulate the nuclease activity of DUBs
UCH	Coiled coil	Q4D6N2, Q4E391, Q4D1W6, Q4E0V5	Diverse biological functions
Ribosomal S19e	Ribosomal S19e	Q4D6N9, Q4DJY1	Role is not established

Domain name abbreviations used are: WLM- Wss like metalloprotease, ZnFRBZ- Zinc finger domain in Ran-binding proteins, zf-RanBP- Zinc finger domain in Ran BP2 binding protein, OTU- Ovarian tumour domain, JAB-Jun kinase activation domain binding protein, PUB- Protein module of unknown function, UCH- Ubiquitin carboxyl-terminal hydrolases, DUSP-Domain present in ubiquitin-

specific proteases, USP7 C2- Ubiquitin specific protease 7 C terminal, USP7 ICP0 bdg- Ubiquitin specific protease 7 ICP0 bdg, zfUBP- Ubiquitin Carboxyl-terminal hydrolase-like zinc finger ,UBA- Ubiquitin associated domain, UBQ- Ubiquitin, SCOP- Superfamily WD40 like repeat , EXOIII- Exonuclease domain.

** Some of the UniProt ids are present in more than one domain category as they are comprised of multiple domains*

Supplementary Table S4: **The complete list of all the predicted proteins localization in different cellular compartments.** UniProt id and cell organelles are given.

**Cytoplasm**	**Nucleus**	**Mitochondria**	**Cytoskeleton**	**Plasma membrane**	**Membrane**	**Peroxisome**
Q4CQH2	Q4D1I0	Q4CMJ7	Q4CKV4	Q4CT25	Q4CPT2	Q4DJF2
Q4CTM0	Q4D5L7	Q4CNA5	Q4CUC6	Q4DC93	Q4D328	
Q4CUS2	Q4D662	Q4CTT9	Q4D7U8	Q4DW46		
Q4CV58	Q4D6F3	Q4CUI7	Q4D821	Q4E0X7		
Q4D029	Q4D6N2	Q4CXY4	Q4DAL0	Q4E2K5		
Q4D0P2	Q4D6N9	Q4D1W6	Q4DCS7			
Q4D3X7	Q4D8Z5	Q4D3R6	Q4DD96			
Q4D547	Q4DB05	Q4D5K1	Q4DFC9			
Q4D5C1	Q4DB31	Q4D5L1	Q4DT67			
Q4D6X2	Q4DCF4	Q4D6U5	Q4DTJ5			
Q4DFD2	Q4DF80	Q4DDC1	Q4DXI0			
Q4DG61	Q4DFR6	Q4DDT1	Q4E4I8			

Q4DHT9	Q4DFS1	Q4DJY1	Q4DFC9			
Q4DJ21	Q4DG39	Q4DKG4	Q4E680			
Q4DM33	Q4DGF2	Q4DNJ4				
Q4DNK3	Q4DI78	Q4DNK8				
Q4DX31	Q4DIY7	Q4DPR2				
Q4DYG8	Q4DKZ7	Q4DS35				
Q4E4Q7	Q4DL73	Q4DW10				
	Q4DLU8	Q4DWK7				
	Q4DMB6	Q4DWL2				
	Q4DPJ5	Q4E0V5				
	Q4DPM7	Q4E0K4				
	Q4DPS6	Q4E560				
	Q4DR65	Q4E565				
	Q4DRN4	Q4CKL0				
	Q4DRP0	Q4CRP5				
	Q4DSB2	Q4CX33				
	Q4DSZ1	Q4CY74				
	Q4DVD1	Q4CZE8				

	Q4DW87	Q4D0Q6				
	Q4DWH5	Q4D2A5				
	Q4DZ28	Q4D4T1				
	Q4DZU8	Q4DAB5				
	Q4E0W2	Q4DG19				
	Q4E259	Q4DMK1				
	Q4E391	Q4DNX6				
	Q4E407	Q4DP57				
	Q4E413	Q4DYE1				
	Q4E4N3	Q4E0S6				
	Q4E655	Q4E398				
	Q4DDL9	Q4E3Z8				
	Q4DYL1	Q4E4T0				
	Q4DCC7	Q4E5S0				
	Q4DH98	Q4DFK1				
	Q4CK94					
	Q4CKK6					
	Q4CPI2					

	Q4CQA0					
	Q4CQT6					
	Q4CS38					
	Q4CSC6					
	Q4CSJ0					
	Q4CTT7					
	Q4CUC0					
	Q4CVD0					
	Q4CYA6					
	Q4CZ35					
	Q4D018					
	Q4D0S8					
	Q4D0X0					

Supplementary Table S5: **The complete list of all the predicted proteins secreted and localized in more than one cellular compartments.** UniProt id and cell organelles are given

**Cytoplasm, Nucleus**	**Cytoplasm, Mitochondria**	**Mitochondria (membrane)**	**Secreted**	**Nucleus, Secreted**	**Mitochondria, Secreted**
Q4CK98	Q4CPQ0	Q4CSG2	Q4CR46	Q4DQZ2	Q4CYA0
Q4DPZ0	Q4D313	Q4DYQ0	Q4D897	Q4DXR1	Q4CRZ3
Q4D072			Q4E1T1	Q4DAZ0	Q4DLE5
Q4E1P7			Q4E4A5	Q4DWV9	Q4CL15
				Q4DXD8	Q4E3Q8
					Q4CYB2

Supplementary Table S6: **The complete list of all the proteins upregulated and downregulated using expression data analysis at four life cycle stages using a cutoff value of 1.2.** UniProt id and stages are given. Abbreviations used: a-amastigote, t-trypomastigote, e-epimastigote, m-metacyclics.

**Stages**	**a**	**t**	**e**	**m**
**Upregulated Gene (Uniprot Ids)**	Q4D5L7	Q4CS38	Q4D3X5	Q4E1T1
	Q4D6X2	Q4DL11	Q4DFS1	Q4CUC6
	Q4D7U8	Q4DWL2	Q4DIY7	Q4CUS2

	Q4D897	Q4E0W2	Q4DYQ0	Q4DWH5
	Q4DMB6	Q4E5S0		Q4CYA0
	Q4DSZ1			Q4CZ35
				Q4D0Q6
				Q4D547
				Q4D5K1
				Q4D8Z5
				Q4DFK1
				Q4DKG4
				Q4DNJ4

**Stages**	**a**	**t**	**e**	**m**
**Downregulated Gene (Uniprot Ids)**	Q4CMJ7	Q4D3X7	Q4CVD0	Q4CYA6
	Q4CTT9	Q4DW10	Q4CPT2	Q4D4T1
	Q4CUS2	Q4DXI0	Q4CS38	Q4D6X2
	Q4CYA0		Q4DAL0	Q4DFD2
	Q4D0Q6		Q4DPM7	Q4DG61

	Q4D6U5		Q4DR65	Q4DSZ1
	Q4DB31		Q4DWK7	Q4E398
	Q4DC93		Q4DZU8	Q4E413
	Q4DHT9		Q4E0X7	
	Q4DKG4		Q4E1P7	
	Q4DS35		Q4E391	
	Q4DWH5		Q4E4A5	
	Q4DYE1		Q4E4N3	
			Q4E5S0	

References:

1 Durocher, D. & Jackson, S. P. The FHA domain. *FEBS letters* **513**, 58-66 (2002).

2 Bienz, M. The PHD finger, a nuclear protein-interaction domain. *Trends in biochemical sciences* **31**, 35-40 (2006).

3 Platta, H. W. *et al.* Pex2 and pex12 function as protein-ubiquitin ligases in peroxisomal protein import. *Molecular and cellular biology* **29**, 5505-5516 (2009).

4 Langelier, M.-F. & Pascal, J. M. PARP-1 mechanism for coupling DNA damage detection to poly (ADP-ribose) synthesis. *Current opinion in structural biology* **23**, 134-143 (2013).

5 Klose, R. J., Kallin, E. M. & Zhang, Y. JmjC-domain-containing proteins and histone demethylation. *Nature Reviews Genetics* **7**, 715-727 (2006).

6 Vander Kooi, C. W. *et al.* The Prp19 WD40 domain contains a conserved protein interaction region essential for its function. *Structure* **18**, 584-593 (2010).

7 Reyes-Turcu, F. E., Ventii, K. H. & Wilkinson, K. D. Regulation and cellular roles of ubiquitin-specific deubiquitinating enzymes. *Annual review of biochemistry* **78**, 363-397 (2009).

8 Perusina Lanfranca, M., Mostafa, H. H. & Davido, D. J. HSV-1 ICP0: an E3 ubiquitin ligase that counteracts host intrinsic and innate immunity. *Cells* **3**, 438-454 (2014).
